# Supplementary material for: Activating an Interleukin-4-FLT3-STAT6 axis in Multipotent Progenitors Restores Lymphopoiesis in Inflammation and Aging
Source: Immunity. Author manuscript; Available in PMC 2026 Jul 27. (PMC13404287; doi:10.1016/j.immuni.2026.04.018)
Supplement: Supplemental Information [file NIHMS2194208-supplement-Supplemental_Information.pdf]

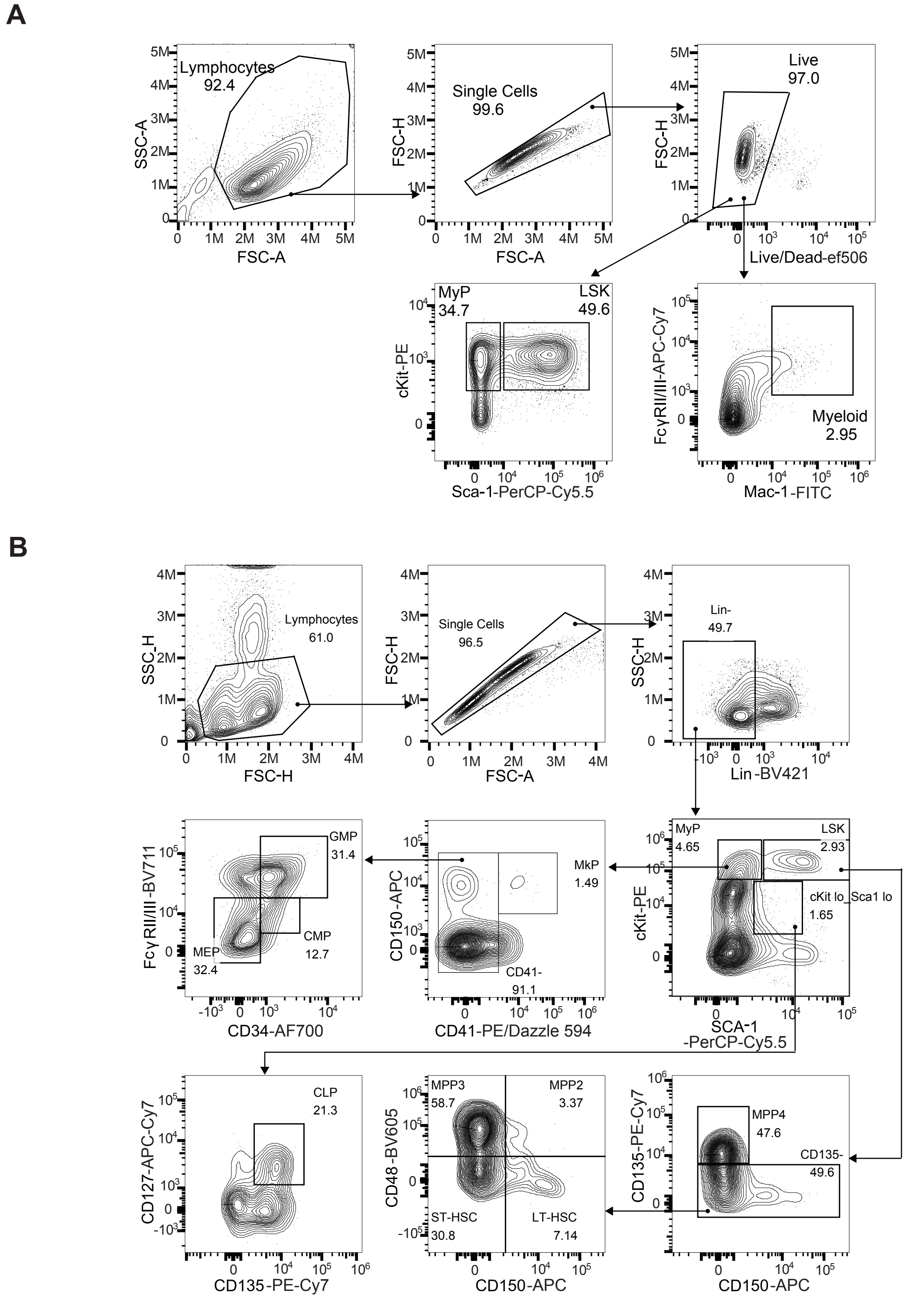

A
Lymphocytes
92.4
SSC-A
FSC-A
Single Cells
99.6
FSC-H
FSC-A
Live
97.0
FSC-H
Live/Dead-ef506
MyP
34.7
LSK
49.6
cKit-PE
Sca-1-PerCP-Cy5.5
FcγRII/III-APC-Cy7
Myeloid
2.95
Mac-1-FITC
B
Lymphocytes
61.0
SSC_H
FSC-H
Single Cells
96.5
FSC-H
FSC-A
Lin-
49.7
SSC-H
Lin-BV421
GMP
31.4
CMP
12.7
MEP
32.4
FcγRII/III-BV711
CD34-AF700
MkP
1.49
CD41-
91.1
CD150-APC
CD41-PE/Dazzle 594
MyP
4.65
LSK
2.93
cKit lo_Sca1 lo
1.65
cKit-PE
SCA-1
-PerCP-Cy5.5
CLP
21.3
CD127-APC-Cy7
CD135-PE-Cy7
MPP3
58.7
MPP2
3.37
ST-HSC
30.8
LT-HSC
7.14
CD48-BV605
CD150-APC
MPP4
47.6
CD135-
49.6
CD135-PE-Cy7
CD150-APC

Figure S1. Gating strategy (Related to Figure 1)

A, Representative gating strategy for *in vitro* myeloid cells differentiation assay. **B**, Representative gating strategy for BM progenitor populations.

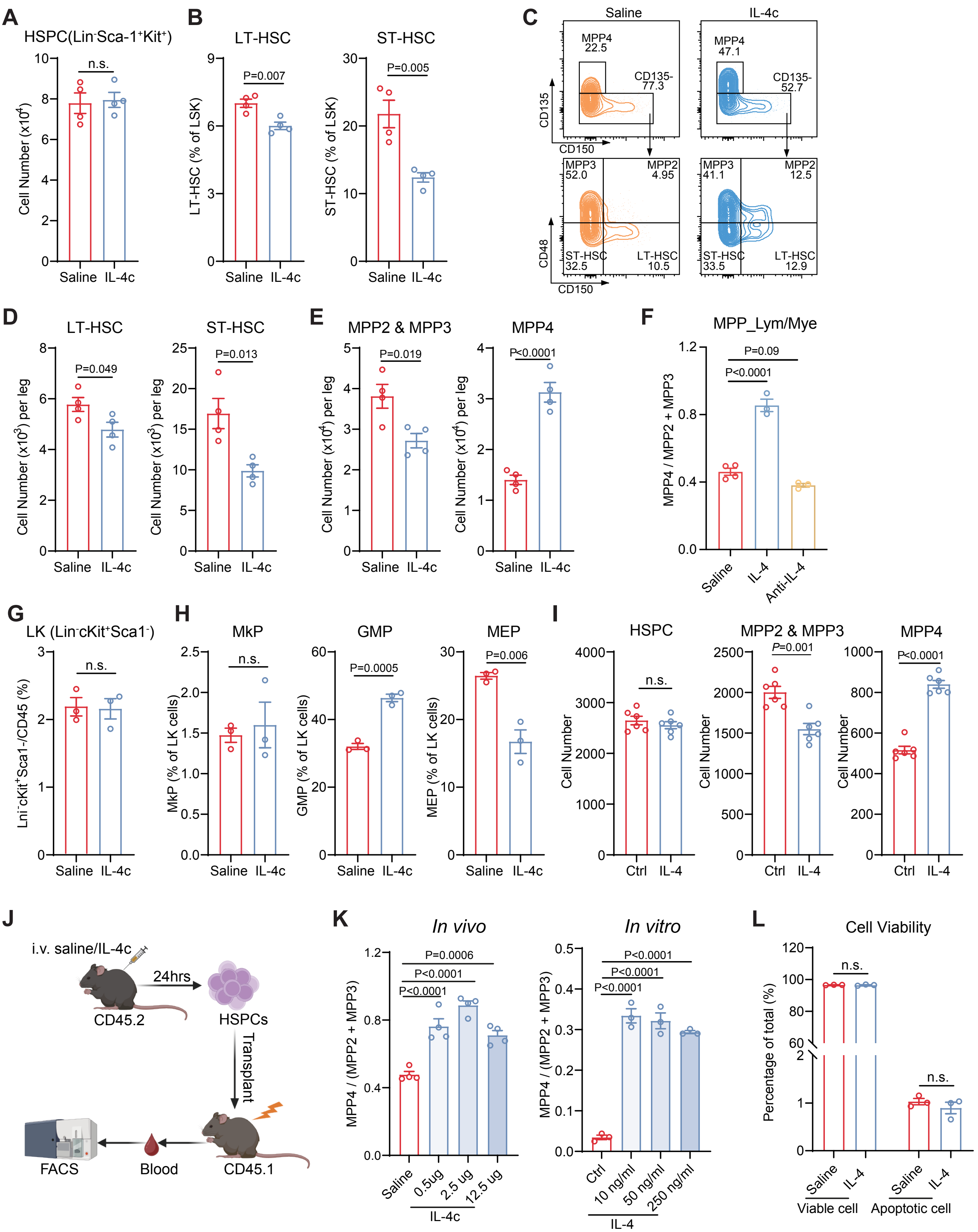

A
HSPC(Lin-Sca-1+Kit+)
n.s.
Cell Number (x10^4)
Saline IL-4c
B
LT-HSC
P=0.007
LT-HSC (% of LSK)
ST-HSC
P=0.005
ST-HSC (% of LSK)
C
Saline
IL-4c
MPP4 22.5
CD135- 77.3
MPP4 47.1
CD135- 52.7
CD135
CD150
MPP3 52.0
MPP2 4.95
ST-HSC 32.5
LT-HSC 10.5
MPP3 41.1
MPP2 12.5
ST-HSC 33.5
LT-HSC 12.9
CD48
D
LT-HSC
P=0.049
ST-HSC
P=0.013
Cell Number (x10^3) per leg
E
MPP2 & MPP3
P=0.019
MPP4
P<0.0001
Cell Number (x10^4) per leg
F
MPP_Lym/Mye
P=0.09
P<0.0001
MPP4 / MPP2 + MPP3
Saline IL-4 Anti-IL-4
G
LK (Lin-cKit+Sca1-)
n.s.
Lni-cKit+Sca1-/CD45 (%)
H
MkP
n.s.
MkP (% of LK cells)
GMP
P=0.0005
GMP (% of LK cells)
MEP
P=0.006
MEP (% of LK cells)
I
HSPC
n.s.
MPP2 & MPP3
P=0.001
MPP4
P<0.0001
Cell Number
Ctrl IL-4
J
i.v. saline/IL-4c
24hrs
CD45.2
HSPCs
Transplant
FACS
Blood
CD45.1
K
In vivo
P=0.0006
P<0.0001
P<0.0001
MPP4 / (MPP2 + MPP3)
Saline 0.5ug 2.5 ug 12.5 ug
IL-4c
In vitro
P<0.0001
P<0.0001
P<0.0001
Ctrl 10 ng/ml 50 ng/ml 250 ng/ml
IL-4
L
Cell Viability
n.s.
n.s.
Percentage of total (%)
Saline IL-4
Viable cell
Apoptotic cell

Figure S2. Effects of IL-4 treatment on hematopoietic stem and progenitor cells, and downstream cells (Related to Figure 1)

A, Absolute numbers of the BM HSPCs from mice with or without IL-4c treatment (n=4). **B**, Percentage of BM LT-HSC and ST-HSC in HSPCs from mice with or without IL-4c treatment (n=4). **C**, Representative FACS plots of LT-HSC, ST-HSC, MPP2, MPP3 and MPP4 within HSPCs from mice with or without IL-4c treatment. **D-E**, Absolute numbers of LT-HSC and ST-HSC (**D**) as well as MPP2 & MPP3 and MPP4 (**E**) per leg with or without IL-4c treatment *in vivo* (n=4). **F**, The BM MPP_Lym/Mye ratio of WT mice treated with 2.5 ug IL-4 alone or 12.5 ug anti-IL-4 antibody alone for 24 hours (n=3-4). **G-H**, Percentage of BM LK populations (**G**), MkP, GMP and MEP (**H**) from mice with or without IL-4c treatment *in vivo* (n=3). **I**, Absolute numbers of the indicated populations (HPSCs, MPP2 & MPP3 and MPP4) with or without 50 ng/ml IL-4 treatment *in vitro* (n=6). **J**, Schematic of transplantation of HSPCs from IL-4c treated or untreated mice in lethally irradiated recipients. **K**, The MPP_Lym/Mye ratio after different doses of IL-4 treatment from *in vivo* (Left panel, n=4) and *in vitro* (Right panel, n=3). **L**, HSPC viability analysis after IL-4 treatment for 24 hours *in vitro* (n=3). All data represent means ± s.e.m. Statistical significance was determined by one-way ANOVA with Tukey's multiple-comparisons test (**K**) or unpaired two-tailed Student's t-test (**A-B**, **D-I** and **L**).

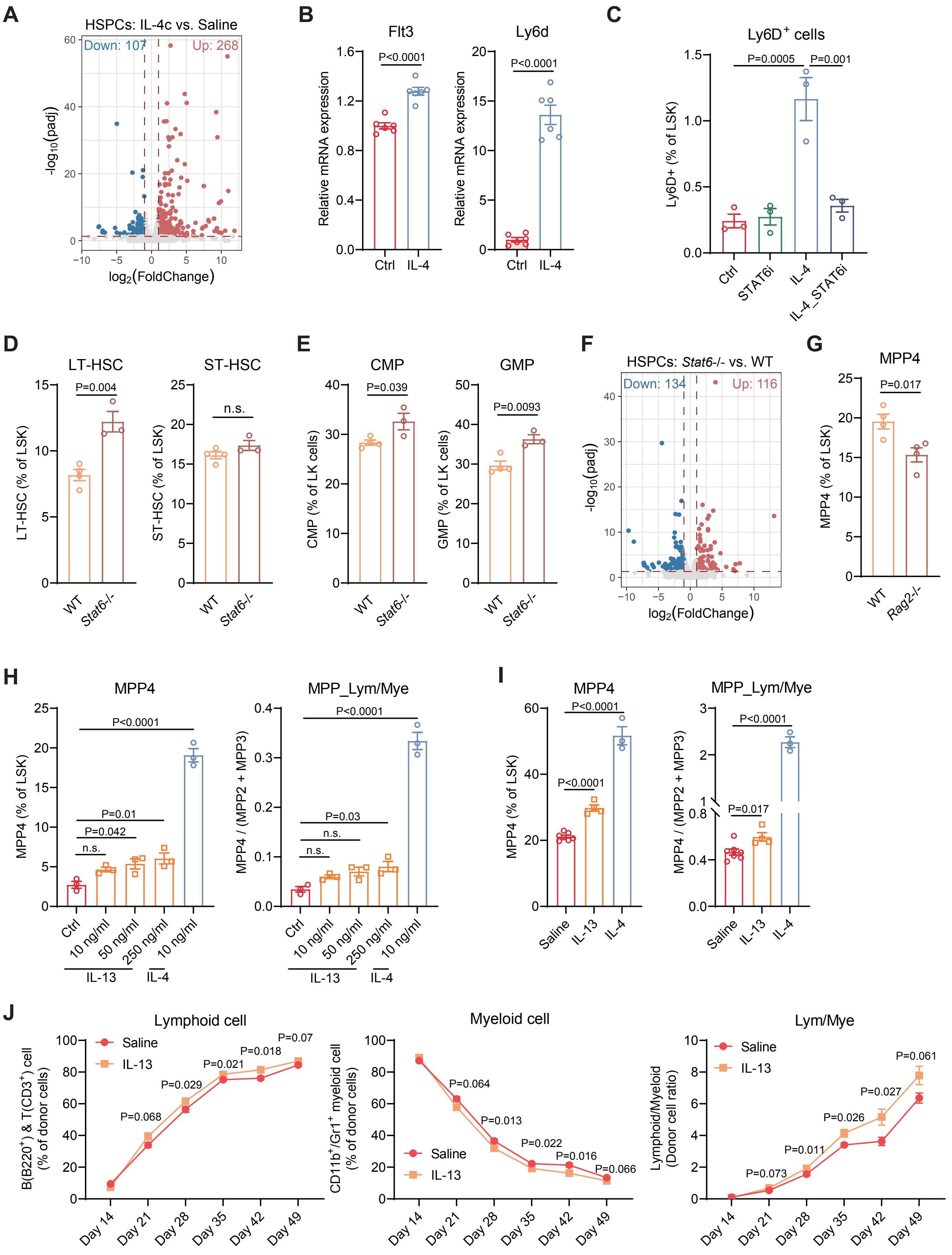
A
HSPCs: IL-4c vs. Saline
Down: 107
Up: 268
-log10(padj)
log2(FoldChange)
B
Flt3
Ly6d
P<0.0001
P<0.0001
Relative mRNA expression
Ctrl
IL-4
C
Ly6D+ cells
P=0.0005
P=0.001
Ly6D+ (% of LSK)
Ctrl
STAT6i
IL-4
IL-4_STAT6i
D
LT-HSC
ST-HSC
P=0.004
n.s.
LT-HSC (% of LSK)
ST-HSC (% of LSK)
WT
Stat6-/-
E
CMP
GMP
P=0.039
P=0.0093
CMP (% of LK cells)
GMP (% of LK cells)
F
HSPCs: Stat6-/- vs. WT
Down: 134
Up: 116
G
MPP4
P=0.017
MPP4 (% of LSK)
WT
Rag2-/-
H
MPP4
MPP_Lym/Mye
P<0.0001
P=0.01
P=0.042
P=0.03
n.s.
MPP4 (% of LSK)
MPP4 / (MPP2 + MPP3)
Ctrl
10 ng/ml
50 ng/ml
250 ng/ml
IL-13
IL-4
I
MPP4
MPP_Lym/Mye
P<0.0001
P=0.017
Saline
IL-13
IL-4
J
Lymphoid cell
Myeloid cell
Lym/Mye
Saline
IL-13
B(B220+) & T(CD3+) cell (% of donor cells)
CD11b+/Gr1+ myeloid cell (% of donor cells)
Lymphoid/Myeloid (Donor cell ratio)
P=0.068
P=0.029
P=0.021
P=0.018
P=0.07
P=0.064
P=0.013
P=0.022
P=0.016
P=0.066
P=0.073
P=0.011
P=0.026
P=0.027
P=0.061
Day 14
Day 21
Day 28
Day 35
Day 42
Day 49

Figure S3. The STAT6 activation regulates genes important for lymphopoiesis (Related to Figure 2)

A, Volcano plot showing the DEGs in HSPCs from IL-4c-treated versus saline-treated mice. **B**, RT–qPCR analysis of *Flt3* and *Ly6d* gene expression in isolated HSPCs treated with or without 50 ng/ml IL-4 for 24 hours *in vitro* (n=6). **C**, Percentage of Ly6D$^+$ cells in isolated HSPCs with or without STAT6 inhibitor treatment *in vitro* (n=3). **D**, Percentage of BM LT-HSC and ST-HSC in HSPCs from WT and *Stat6*$^{-/-}$ mice (n=3-4). **E**, Percentage of BM CMP and GMP in LK from WT and *Stat6*$^{-/-}$ mice (n=3-4). **F**, Volcano plot showing the DEGs in HSPCs from *Stat6*$^{-/-}$ versus WT mice. **G**, Percentage of BM MPP4 in HSPCs from WT and *Rag2*$^{-/-}$ mice (n=4). **H**, Percentage of MPP4 subpopulations within isolated HSPCs and the MPP_Lym/Mye ratio, with or without 24 hours treatment using different concentration of IL-13 or IL-4 *in vitro* (n=3). **I**, Percentage of MPP4 subpopulations within HSPCs and the MPP_Lym/Mye ratio from WT mice with or without IL-13 or IL-4c injections (n=3-6). **J**, Transplantation of MPPs from IL-4c treated or untreated mice to lethally irradiated recipients: Donor-derived lymphoid cells, myeloid cells and lymphoid-to-myeloid ratio in peripheral blood at the indicated days post-transplantation (n=5). All data represent means ± s.e.m. Statistical significance was determined by one-way ANOVA with Tukey's multiple-comparisons test (**C, H** and **I**) or unpaired two-tailed Student's t-test (**B, D-E, G** and **J**).

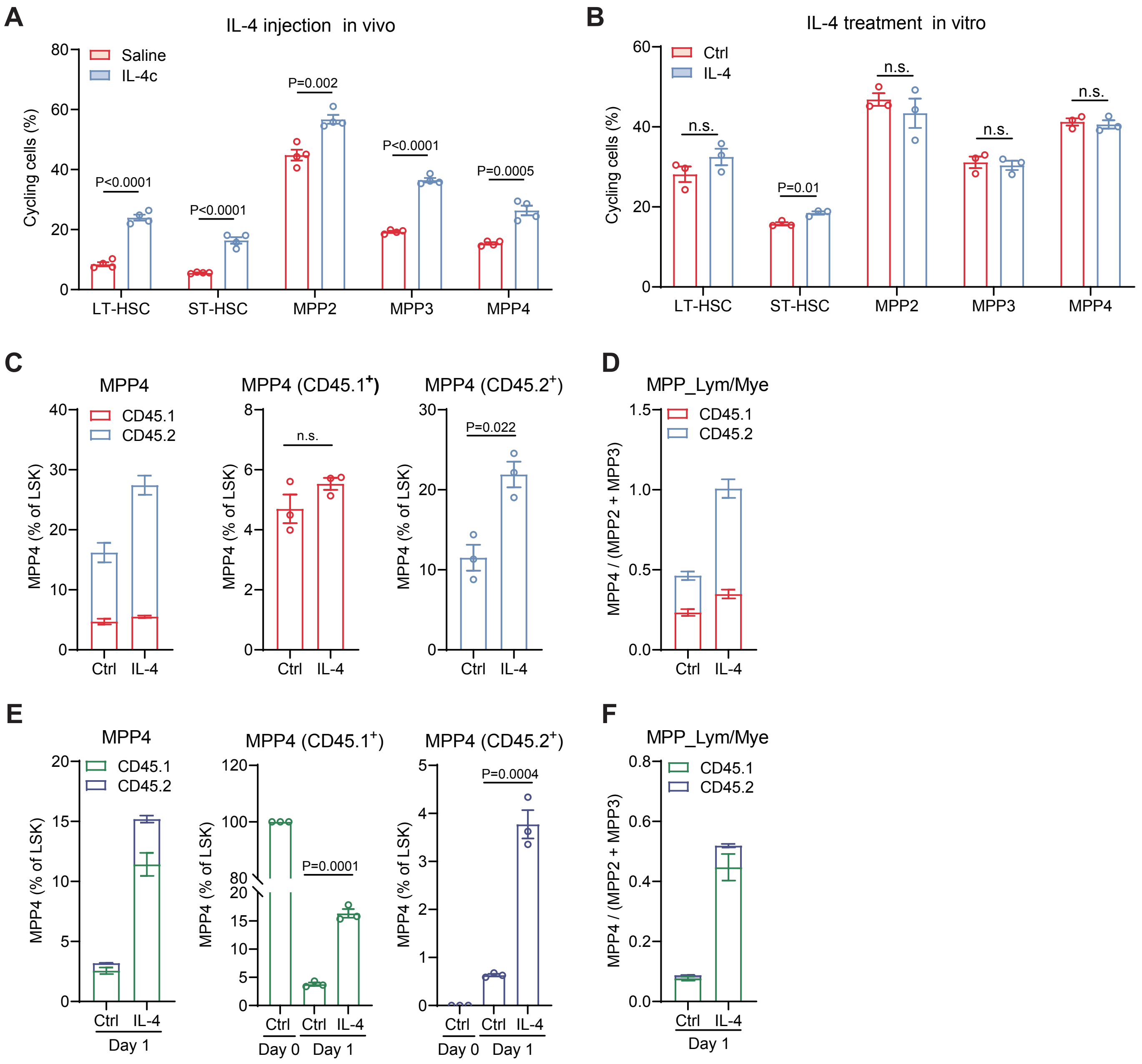

A
IL-4 injection in vivo
Saline
IL-4c
Cycling cells (%)
P<0.0001
P<0.0001
P=0.002
P<0.0001
P=0.0005
LT-HSC
ST-HSC
MPP2
MPP3
MPP4
B
IL-4 treatment in vitro
Ctrl
IL-4
Cycling cells (%)
n.s.
P=0.01
n.s.
n.s.
n.s.
LT-HSC
ST-HSC
MPP2
MPP3
MPP4
C
MPP4
CD45.1
CD45.2
MPP4 (% of LSK)
Ctrl
IL-4
MPP4 (CD45.1+)
n.s.
MPP4 (CD45.2+)
P=0.022
D
MPP_Lym/Mye
CD45.1
CD45.2
MPP4 / (MPP2 + MPP3)
Ctrl
IL-4
E
MPP4
CD45.1
CD45.2
MPP4 (% of LSK)
Ctrl
IL-4
Day 1
MPP4 (CD45.1+)
P=0.0001
Ctrl
Ctrl
IL-4
Day 0
Day 1
MPP4 (CD45.2+)
P=0.0004
Ctrl
Ctrl
IL-4
Day 0
Day 1
F
MPP_Lym/Mye
CD45.1
CD45.2
MPP4 / (MPP2 + MPP3)
Ctrl
IL-4
Day 1

Figure S4. Effects of IL-4 treatment on HSPC proliferation and differentiation as well as MPP transition (Related to Figure 3)

A-B, Percentage of cycling cells of indicated populations with or without IL-4c treatment *in vivo* (**A**) (n=4) or *in vitro* (**B**) (n=3). **C-D**, Percentage of MPP4 within co-cultured CD45.1 HSCs and CD45.2 MPPs (**C**), and the MPP_Lym/Mye ratio (**D**), with or without treatment using 50 ng/mL IL-4 for 24 hours *in vitro* (n=3). **E-F**, Percentage of MPP4 within co-cultured CD45.1 MPP4 and CD45.2 MPP2 & 3 (**E**), and the MPP_Lym/Mye ratio (**F**), with or without treatment using 50 ng/mL IL-4 for 24 hours *in vitro* (n=3). All data represent means ± s.e.m. Statistical significance was determined by unpaired two-tailed Student's t-test (**A-F**).

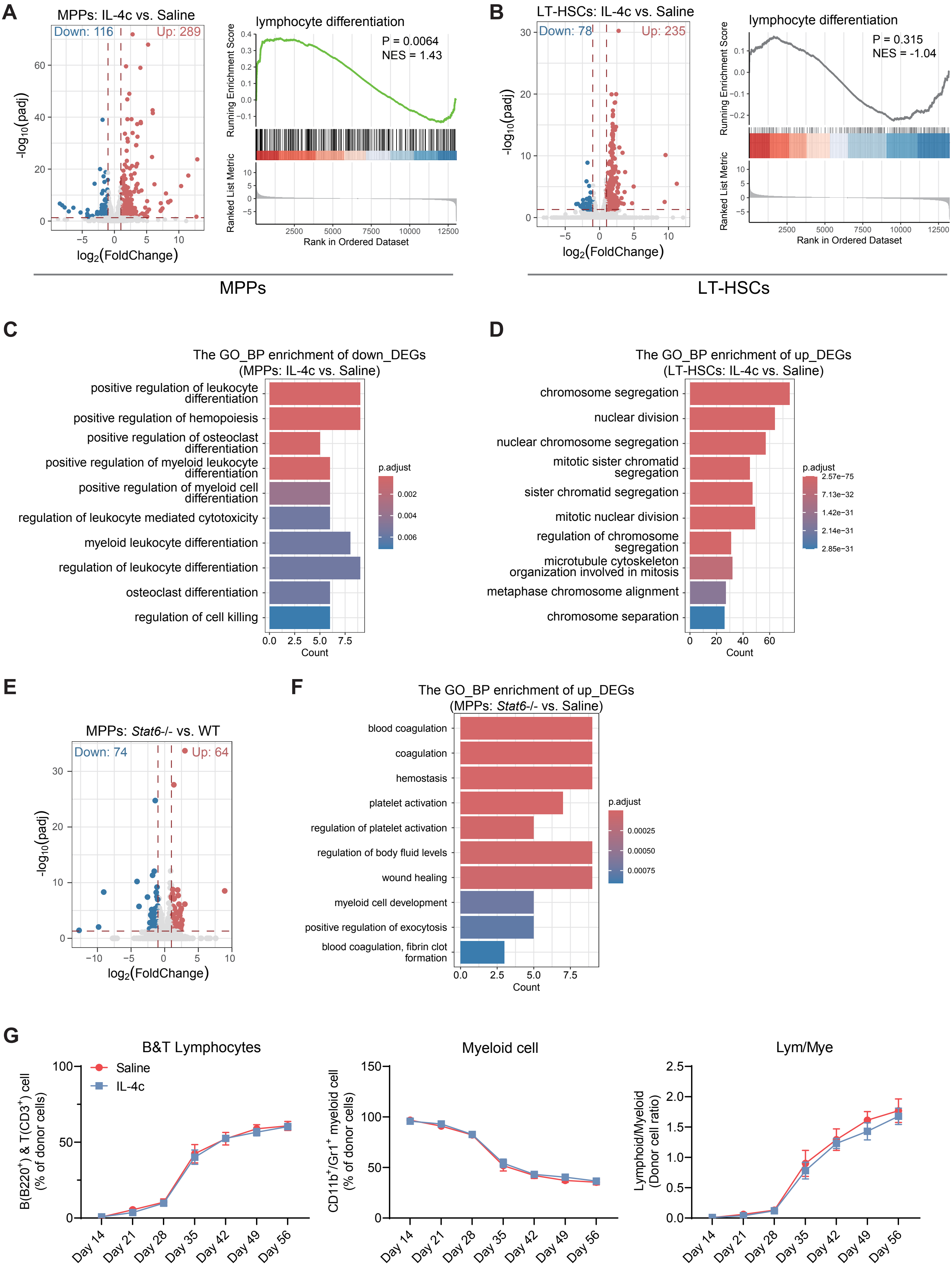

A
MPPs: IL-4c vs. Saline
Down: 116
Up: 289
-log10(padj)
log2(FoldChange)
lymphocyte differentiation
P = 0.0064
NES = 1.43
Running Enrichment Score
Ranked List Metric
Rank in Ordered Dataset
B
LT-HSCs: IL-4c vs. Saline
Down: 78
Up: 235
lymphocyte differentiation
P = 0.315
NES = -1.04
MPPs
LT-HSCs
C
The GO_BP enrichment of down_DEGs
(MPPs: IL-4c vs. Saline)
positive regulation of leukocyte differentiation
positive regulation of hemopoiesis
positive regulation of osteoclast differentiation
positive regulation of myeloid leukocyte differentiation
positive regulation of myeloid cell differentiation
regulation of leukocyte mediated cytotoxicity
myeloid leukocyte differentiation
regulation of leukocyte differentiation
osteoclast differentiation
regulation of cell killing
p.adjust
Count
D
The GO_BP enrichment of up_DEGs
(LT-HSCs: IL-4c vs. Saline)
chromosome segregation
nuclear division
nuclear chromosome segregation
mitotic sister chromatid segregation
sister chromatid segregation
mitotic nuclear division
regulation of chromosome segregation
microtubule cytoskeleton organization involved in mitosis
metaphase chromosome alignment
chromosome separation
p.adjust
2.57e−75
7.13e−32
1.42e−31
2.14e−31
2.85e−31
Count
E
MPPs: Stat6-/- vs. WT
Down: 74
Up: 64
-log10(padj)
log2(FoldChange)
F
The GO_BP enrichment of up_DEGs
(MPPs: Stat6-/- vs. Saline)
blood coagulation
coagulation
hemostasis
platelet activation
regulation of platelet activation
regulation of body fluid levels
wound healing
myeloid cell development
positive regulation of exocytosis
blood coagulation, fibrin clot formation
p.adjust
Count
G
B&T Lymphocytes
Saline
IL-4c
B(B220+) & T(CD3+) cell (% of donor cells)
Myeloid cell
CD11b+/Gr1+ myeloid cell (% of donor cells)
Lym/Mye
Lymphoid/Myeloid (Donor cell ratio)
Day 14
Day 21
Day 28
Day 35
Day 42
Day 49
Day 56

Figure S5. Transcriptomic analysis of MPPs and LT-HSCs after IL-4 treatment (Related to Figure 3)

A-B, Volcano plot (left) showing the DEGs and GSEA enrichment (right) showing the "lymphoid differentiation" pathways in MPPs (**A**) or LT-HSCs (**B**) from IL-4c-treated versus saline-treated mice. **C-D**, GO pathway enrichment analyses of genes significantly downregulated in MPPs (**C,** log_2FoldChange < −1, Padj<0.05) and upregulated in LT-HSCs (**D,** log_2FoldChange > 1, Padj<0.05) from IL-4c-treated versus saline-treated mice. **E**, Volcano plot showing the DEGs in MPPs from *Stat6*$^{-/-}$ versus WT mice. **F**. GO pathway enrichment analyses of genes significantly upregulated in MPPs from *Stat6*$^{-/-}$ mice versus WT mice. (log_2FoldChange > 1, Padj<0.05). **G**, Transplantation of LT-HSCs from IL-4c treated or untreated mice to lethally irradiated recipients: Donor-derived lymphoid cells (**G**, **left**), myeloid cells (**G**, **middle**) and lymphoid-to-myeloid ratio (**G**, **right**) in peripheral blood at the indicated days post-transplantation (n=5).

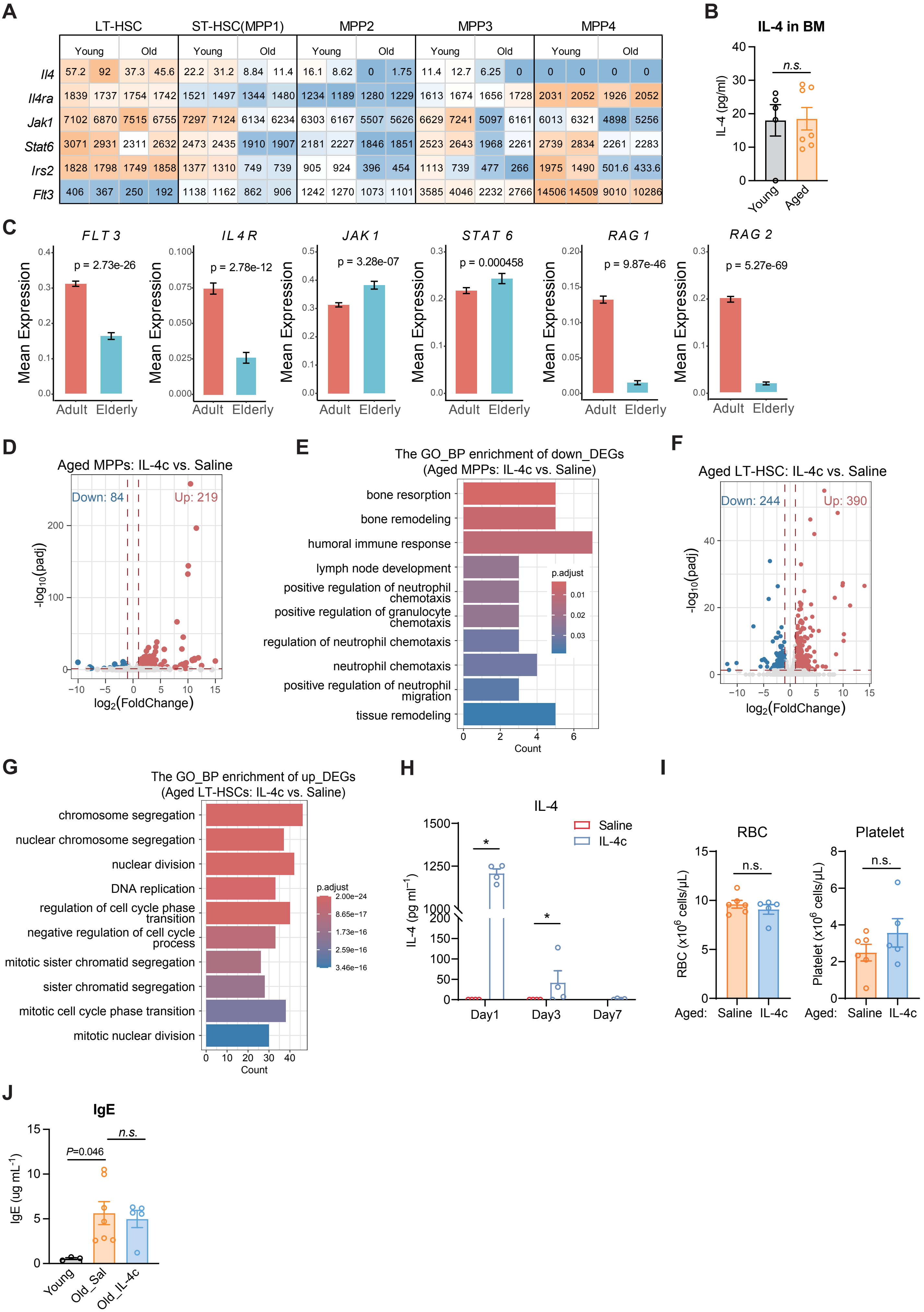
A
LT-HSC
ST-HSC(MPP1)
MPP2
MPP3
MPP4
Young
Old
Il4 57.2 92 37.3 45.6 22.2 31.2 8.84 11.4 16.1 8.62 0 1.75 11.4 12.7 6.25 0 0 0 0 0
Il4ra 1839 1737 1754 1742 1521 1497 1344 1480 1234 1189 1280 1229 1613 1674 1656 1728 2031 2052 1926 2052
Jak1 7102 6870 7515 6755 7297 7124 6134 6234 6303 6167 5507 5626 6629 7241 5097 6161 6013 6321 4898 5256
Stat6 3071 2931 2311 2632 2473 2435 1910 1907 2181 2227 1846 1851 2523 2643 1968 2261 2739 2834 2261 2283
Irs2 1828 1798 1749 1858 1377 1310 749 739 905 924 396 454 1113 739 477 266 1975 1490 501.6 433.6
Flt3 406 367 250 192 1138 1162 862 906 1242 1270 1073 1101 3585 4046 2232 2766 14506 14509 9010 10286
B
IL-4 in BM
IL-4 (pg/ml)
n.s.
Young
Aged
C
FLT3
p = 2.73e-26
IL4R
p = 2.78e-12
JAK1
p = 3.28e-07
STAT6
p = 0.000458
RAG1
p = 9.87e-46
RAG2
p = 5.27e-69
Mean Expression
Adult
Elderly
D
Aged MPPs: IL-4c vs. Saline
Down: 84
Up: 219
-log10(padj)
log2(FoldChange)
E
The GO_BP enrichment of down_DEGs
(Aged MPPs: IL-4c vs. Saline)
bone resorption
bone remodeling
humoral immune response
lymph node development
positive regulation of neutrophil chemotaxis
positive regulation of granulocyte chemotaxis
regulation of neutrophil chemotaxis
neutrophil chemotaxis
positive regulation of neutrophil migration
tissue remodeling
p.adjust
Count
F
Aged LT-HSC: IL-4c vs. Saline
Down: 244
Up: 390
-log10(padj)
log2(FoldChange)
G
The GO_BP enrichment of up_DEGs
(Aged LT-HSCs: IL-4c vs. Saline)
chromosome segregation
nuclear chromosome segregation
nuclear division
DNA replication
regulation of cell cycle phase transition
negative regulation of cell cycle process
mitotic sister chromatid segregation
sister chromatid segregation
mitotic cell cycle phase transition
mitotic nuclear division
p.adjust
2.00e−24
8.65e−17
1.73e−16
2.59e−16
3.46e−16
Count
H
IL-4
Saline
IL-4c
IL-4 (pg ml−1)
*
Day1
Day3
Day7
I
RBC
Platelet
RBC (x10^6 cells/μL)
Platelet (x10^6 cells/μL)
n.s.
Aged:
Saline
IL-4c
J
IgE
IgE (ug mL−1)
P=0.046
n.s.
Young
Old_Sal
Old_IL-4c

Figure S6. IL-4 effects on hematopoiesis in aged mice (Related to Figure 6)

A, Heatmap of relative RNA expression of IL-4 downstream signaling molecules and *Flt3* in LT-HSC, ST-HSC and different MPPs from both young and aged mice. **B**, IL-4 level in young and aged BM fluid samples (n=5-7). **C**, IL-4 downstream signaling gene expression in human HSPCs from adult (25–53 years) and elderly (62–77 years) donors. **D**, Volcano plot showing the DEGs in MPPs from IL-4c-treated versus saline-treated aged mice. **E**, GO pathway enrichment analyses of genes significantly downregulated in MPPs from IL-4c-treated versus saline-treated aged mice (log_2FoldChange < -1, Padj<0.05). **F**, Volcano plot showing the DEGs in LT-HSCs from IL-4c-treated versus saline-treated aged mice. **G**, GO pathway enrichment analyses of genes significantly upregulated in LT-HSCs from IL-4c-treated versus saline-treated aged mice (log_2FoldChange > 1, Padj<0.05). **H**, IL-4 levels in serum following IL-4c injection, measured at indicated time points (n=4). **I**, Complete blood counting analysis of red blood cell (RBC) and platelet from aged mice with or without long-term IL-4c injections (n=5-6). **J**, IgE level in serum after long-term IL-4 treatment in aged mice (n=3-7). All data represent means ± s.e.m. Statistical significance was determined by one-way ANOVA with Tukey's multiple-comparisons test (**J**) or unpaired two-tailed Student's t-test (**B-C** and **H-I**).

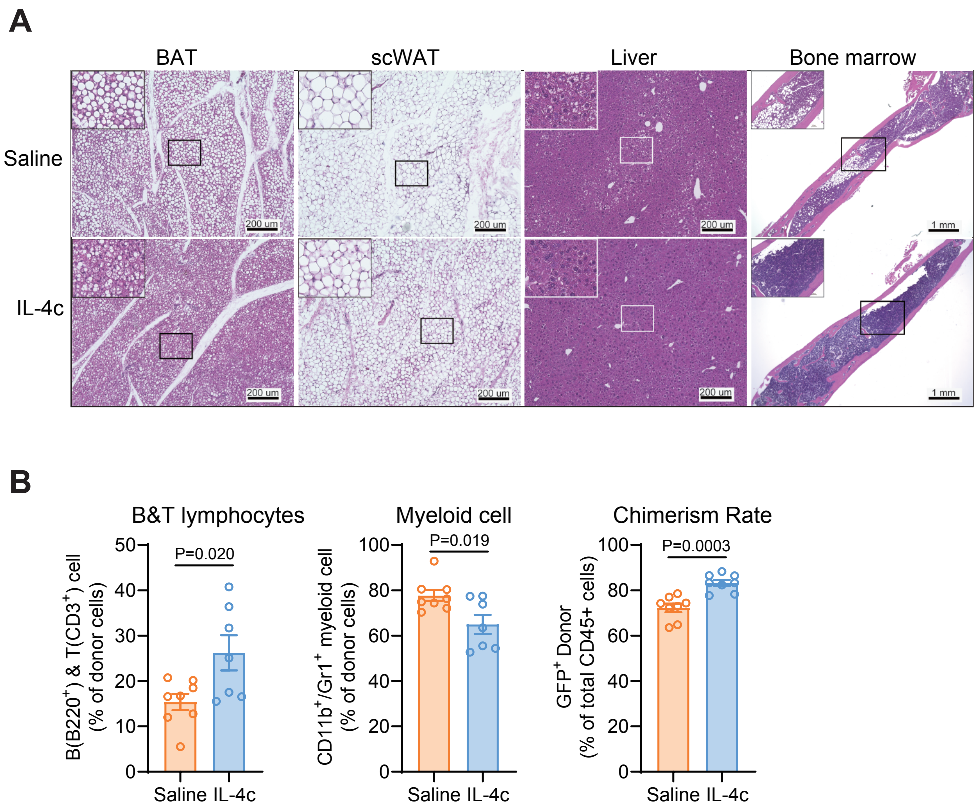

A
BAT
scWAT
Liver
Bone marrow
Saline
IL-4c
200 um
1 mm
B
B&T lymphocytes
Myeloid cell
Chimerism Rate
P=0.020
P=0.019
P=0.0003
B(B220+) & T(CD3+) cell (% of donor cells)
CD11b+/Gr1+ myeloid cell (% of donor cells)
GFP+ Donor (% of total CD45+ cells)
Saline IL-4c

Figure S7. IL-4 treatment mitigates aging-associated decline in tissue function (Related to Figure 7)

A, Representative images of hematoxylin-and-eosin (H&E) -stained sections of BAT (Brown adipose tissue), scWAT (Subcutaneous white adipose tissue), liver and bone marrow of saline or IL-4c treated aged mice. **B**, Donor-derived B and T lymphocytes (left), myeloid cells (middle) and chimerism rate (right) in peripheral blood one-month post-transplantation in aged mice (n=7-8). All data represent means ± s.e.m. Statistical significance was determined by unpaired two-tailed Student's t-test (**B**).
